# S945L-CFTR molecular dynamics, functional characterization and tezacaftor/ivacaftor efficacy *in vivo* and *in vitro* in matched pediatric patient-derived cell models

**DOI:** 10.3389/fped.2022.1062766

**Published:** 2022-11-16

**Authors:** Katelin M. Allan, Miro A. Astore, Laura K. Fawcett, Sharon L. Wong, Po-Chia Chen, Renate Griffith, Adam Jaffe, Serdar Kuyucak, Shafagh A. Waters

**Affiliations:** ^1^ School of Clinical Medicine, Discipline of Paediatrics and Child Health, Faculty of Medicine and Health, UNSW Sydney, Sydney, NSW, Australia; ^2^ Molecular and Integrative Cystic Fibrosis Research Centre, UNSW Sydney, Sydney, NSW, Australia; ^3^ School of Physics, The University of Sydney, Sydney, NSW, Australia; ^4^ Department of Respiratory Medicine, Sydney Children's Hospital, Sydney, NSW, Australia; ^5^ School of Natural Sciences (Chemistry), University of Tasmania, Hobart, TAS, Australia; ^6^ School of Biomedical Sciences, Faculty of Medicine and Health, UNSW Sydney, Sydney, NSW, Australia

**Keywords:** cystic fibrosis, CFTR, modulators, airway epithelial cell models, personalized medicine, molecular dynamics

## Abstract

Cystic Fibrosis (CF) results from over 400 different disease-causing mutations in the CF Transmembrane Conductance Regulator (*CFTR*) gene. These *CFTR* mutations lead to numerous defects in CFTR protein function. A novel class of targeted therapies (CFTR modulators) have been developed that can restore defects in CFTR folding and gating. This study aimed to characterize the functional and structural defects of S945L-CFTR and interrogate the efficacy of modulators with two modes of action: gating potentiator [ivacaftor (IVA)] and folding corrector [tezacaftor (TEZ)]. The response to these modulators *in vitro* in airway differentiated cell models created from a participant with S945L/G542X-CFTR was correlated with *in vivo* clinical outcomes of that participant at least 12 months pre and post modulator therapy. In this participants' airway cell models, CFTR-mediated chloride transport was assessed *via* ion transport electrophysiology. Monotherapy with IVA or TEZ increased CFTR activity, albeit not reaching statistical significance. Combination therapy with TEZ/IVA significantly (*p *= 0.02) increased CFTR activity 1.62-fold above baseline. Assessment of CFTR expression and maturation *via* western blot validated the presence of mature, fully glycosylated CFTR, which increased 4.1-fold in TEZ/IVA-treated cells. The *in vitro* S945L-CFTR response to modulator correlated with an improvement in *in vivo* lung function (ppFEV_1_) from 77.19 in the 12 months pre TEZ/IVA to 80.79 in the 12 months post TEZ/IVA. The slope of decline in ppFEV1 significantly (*p *= 0.02) changed in the 24 months post TEZ/IVA, becoming positive. Furthermore, there was a significant improvement in clinical parameters and a fall in sweat chloride from 68 to 28 mmol/L. The mechanism of dysfunction of S945L-CFTR was elucidated by *in silico* molecular dynamics (MD) simulations. S945L-CFTR caused misfolding of transmembrane helix 8 and disruption of the R domain, a CFTR domain critical to channel gating. This study showed *in vitro* and *in silico* that S945L causes both folding and gating defects in CFTR and demonstrated *in vitro* and *in vivo* that TEZ/IVA is an efficacious modulator combination to address these defects. As such, we support the utility of patient-derived cell models and MD simulations in predicting and understanding the effect of modulators on CFTR function on an individualized basis.

## Introduction

Cystic Fibrosis (CF) is a life-limiting recessive disease caused by mutations in the CF Transmembrane Conductance Regulator (*CFTR*) gene that result in absent or dysfunctional CFTR protein (1). The CFTR protein is a chloride and bicarbonate channel which maintains fluid homeostasis at the apical membrane of epithelial cells in the lungs, pancreas, liver and intestine (2). CFTR is composed of five domains, including two Transmembrane Domains (TMDs) and a Regulatory domain (R domain) (3). In the CFTR open channel state, the TMDs form a pore and allow ions to pass through. In the closed state, the R domain is wedged between the TMDs, preventing them from forming a pore. Phosphorylation of the R domain promotes ATP hydrolysis and increases the open probability of the CFTR channel (4).

Although CF is a monogenic disease, over 2,000 unique mutations have been identified in the *CFTR* gene. To date, 401 of these mutations have been demonstrated to be disease-causing (5), yielding a spectrum of CFTR molecular defects that have been used to classify mutations into six classes (6, 7). CF patients have heterogeneous clinical phenotypes depending on the functional consequence of the mutation(s) on the CFTR protein (8) and disease gene modifiers (9–11). A novel class of targeted therapies (CFTR modulators) have been developed that can restore function to a discrete number of mutant CFTR proteins (12). CFTR modulators currently available have two modes of action: potentiators [e.g., ivacaftor (IVA)] open the CFTR channel (13, 14), while correctors [e.g., lumacaftor (LUM), tezacaftor (TEZ), elexacaftor (ELX)] assist CFTR protein folding and trafficking to the cell surface (15, 16). Clinically approved combination therapies of potentiator and corrector(s) (LUM/IVA, TEZ/IVA, ELX/TEZ/IVA) rescue CFTR function *via* both these mechanisms (17–19).

Patient-derived cell models can be used for *in vitro* CFTR modulator testing as a personalised drug screening platform (20). These platforms can predict the individual's response to treatment and characterise the functional defect caused by *CFTR* mutations. This form of *in vitro* analysis has been permitted by the US Food and Drug Administration (FDA) to facilitate the approval of therapeutics for rare *CFTR* mutations in addition to *in vitro* testing systems using non-primary cells, such as Fisher Rat Thyroid (FRT) cells (21). CFTR-dependent chloride transport assays in patient-derived nasal epithelial cells (22–26) as well as forskolin-induced swelling assays in intestinal organoids (27–31) have been used in a patient-specific manner to predict modulator efficacy for the patient. This utilization of personalized cell models is particularly significant for those patients with rare or poorly characterized mutations that are unable to be tested in clinical trials. Determining the functional response of *CFTR* mutations to modulators with known CFTR correction mechanisms enables characterization of CFTR functional defects and enhances our understanding of CFTR function. In parallel *in silico* Molecular Dynamics (MD) simulations provide a powerful tool to understand the structural basis of CFTR protein function and explore the conformational energy landscape caused by a *CFTR* mutation. The combination approach of using *in vitro* experiments and *in silico* MD simulations has been used previously to characterize rare *CFTR* mutations (30–33). Understanding the functional and structural changes caused by *CFTR* mutations is important for identifying which drug is most likely to benefit a patient.

The S945L (p.Ser945Leu, c.2834C > T) *CFTR* mutation is reported to be carried by 161 patients worldwide (5). The wild-type (WT) amino acid S945 is situated at the end of transmembrane helix 8 (TM8) of the CFTR protein, a region known to contribute to ion selectivity (34) and predicted to bind CFTR potentiators *via* molecular docking (35). IVA, a CFTR potentiator, was approved for use in CF individuals with the S945L mutation by the FDA in 2017. This approval was based on *in vitro* studies using FRT cells expressing S945L in which near-normal CFTR activity was demonstrated in IVA-treated cells (36). However, outside of the US, most patients with the S945L mutation remained without CFTR modulator treatment access until the approval of TEZ/IVA in the US, Canada, Europe, and Australia in 2018/2019. These approvals were based on evidence of TEZ/IVA improving respiratory function (ppFEV_1_) in a Phase III clinical trial of 248 patients 12 years or older heterozygous for F508del, the most common *CFTR* mutation, and a mutation associated with residual CFTR function (13 patients with S945L) (18). In December 2020 in the US, S945L was listed as a mutation which is responsive to treatment by the next generation triple combination CFTR modulator, ELX/TEZ/IVA (37), based on *in vitro* lab data. However, this is only available in Australia to patients with at least one F508del *CFTR* allele.

S945L-CFTR baseline expression and response to IVA has been quantified *via* molecular and functional studies, which describe S945L to interfere with protein folding, channel open probability and chloride conductance (36, 38). However, this work has only been conducted in heterologous systems and since cell background can influence the efficacy of pharmacological response (39), it is important that this is validated in primary cell models. Furthermore, functional testing of S945L response to CFTR corrector is lacking, yet necessary to fully understand the severity of the processing defect incurred by S945L. In addition, there is a gap in the pathophysiological knowledge of how S945L affects CFTR on a structural level. MD simulations of CFTR have previously shown that hydrogen bonds on TM8 stabilise the open state of the channel (40). Since S945L is located on TM8 and therefore in close proximity to these hydrogen bonds, we hypothesised that S945L may cause disruption of the hydrogen bond interactions that stabilise WT-CFTR.

This study reports the clinical outcomes of an individual with CF with the S945L/G542X *CFTR* genotype following initiation of TEZ/IVA therapy. The participant was diagnosed as an infant after presenting with respiratory distress at one month of age. His diagnostic sweat chloride was 74 mmol/L. *Staphylococcus aureus* and *Stenotophomonas maltophilia* were detected on bronchoalveolar lavage at the time of diagnosis. Evidence of bronchiectasis was present on chest x-ray from one year of age. This was confirmed on his first CT at age six for investigation of sputum cultures positive for *Mycobacterium abscessus*. Varicose and cystic bronchiectasis was present, as well as extensive mucous plugging. At the time of enrolment in this study, the participant was eight years old and remained pancreatic sufficient. S945L is a CF-causing *CFTR* mutation that results in pancreatic sufficiency in 60% of individuals when paired with a second CF-causing mutation (5). G542X (p.Gly542X, c.1624G > T), causes a pre-mature stop codon that results in little to no functional protein (41, 42). This enables straightforward functional assessment of S945L in S945L/G542X patient-derived cell models since any CFTR function can be attributed to restoration of the S945L defect. Since CF patients with the S945L mutation are approved for treatment with IVA, TEZ/IVA and ELX/TEX/IVA by the FDA (43), it was expected some benefit would be observed for the participant. The aim of this study was to compare the efficacy of TEZ/IVA for the S945L mutation, *in vitro* and *in vivo*. In addition, we sought to characterise the functional and structural defects caused by the S945L mutation *in vitro*, and *in silico via* MD simulations.

## Materials and methods

### Recruitment and clinical data collection

Following informed consent and prior to treatment with CFTR modulator, the nasal inferior turbinate was brushed, and cells were collected from the enrolled participant with S945L/G542X *CFTR* genotype, as previously described (44). Clinical data were obtained by retrospective review of the participant's medical records. Data was collected from at least 12 months pre initiation of TEZ/IVA treatment. Clinical parameters included spirometry, sweat chloride testing, weight, height and body mass index (BMI). Upon commencing CFTR modulator therapy, the participant underwent prospective clinical monitoring of modulator response and screening for adverse effects for 24 months. Absolute change in clinical parameters was calculated by subtracting the baseline measurement (last measurement pre TEZ/IVA) from the measurement at 12 months post TEZ/IVA initiation. Relative change was calculated as a ratio of the absolute change in comparison to the baseline measurement. Written informed consent was obtained from the participant's legal guardian, with approval from the Sydney Children's Hospital Ethics Review Board (HREC/16/SCHN/120).

### Participant nasal epithelial cell expansion

hNECs were seeded (5,000 cells/cm^2^) into T25 flasks coated with collagen I (Advanced Biomatrix 5015) and pre-seeded with 200,000 irradiated NIH/3T3 feeder cells. hNECs were expanded using conditionally reprogrammed cell (CRC) co-culture with NIH/3T3 feeder cells in house-made CRC culture media supplemented with ROCK (Rho kinase) inhibitor (10 μM) (44–47). After reaching 80%–90% confluence, cells were dissociated *via* differential concentration trypsinisation and seeded for air-liquid interface differentiation.

### Differentiated airway epithelia at air-liquid interface (ALI)

hNECs cells were seeded (150,000 cells/insert) onto 6.5 mm 0.4 µm Transwell porous polyester membranes (Sigma CLS3470) pre-coated with collagen I. Cells were cultured as described previously (30, 44, 47). Briefly, cells were cultured with PneumaCult Ex Plus expansion media (STEMCELL Technologies 05040) until a confluent monolayer was reached (∼4 days). The media was changed to PneumaCult ALI medium (STEMCELL Technologies 05001) to initiate differentiation. Following two days under submerged conditions, an air-liquid interface was established by removing the apical media. Basal media was changed every second day for 21 days.

### Treatment of differentiated airway epithelia with CFTR modulator

Differentiated hNECs were incubated (basal side) with 3 μM VX-809 (LUM, Selleckchem S1565), 5 μM VX-661 (TEZ, Selleckchem S7059) or vehicle control (0.01% DMSO) for 48 h prior to experiments. For ELX/TEZ/IVA treatment, 3 μM VX-445 (ELX, Selleckchem S8851) and 18 μM VX-661 was used. Following 48 h of pre-treatment, differentiated hNECs were mounted in circulating Ussing chambers (see Section “Quantification of CFTR-mediated ion transport in differentiated airway cell models”). 10 μM VX-770 (IVA, Selleckchem S1144) or 0.01% DMSO was added acutely to the apical compartment of the Ussing chamber during CFTR-mediated ion transport assays.

### Quantification of CFTR-mediated ion transport in differentiated airway cell models

Differentiated hNECs were mounted in circulating Ussing chambers (VCC MC8; Physiologic Instruments, San Diego, CA). Short-circuit current (*I*_sc_, µA/cm^2^) was measured under asymmetric chloride (Cl^−^) Ringer's buffer (bicarbonate free) as previously described (47). After recording baseline current for 30 min, cells were sequentially treated with pharmacological compounds: 100 μM apical amiloride to inhibit epithelial sodium channels, 10 μM apical VX-770 (IVA) or 0.01% DMSO (vehicle) to potentiate CFTR-activated currents, 10 μM basal forskolin induce cAMP activation of CFTR, 30 μM apical CFTR_Inh−172_ to inhibit CFTR-specific currents and 100 μM apical ATP to activate calcium-activated chloride currents. Data recordings were obtained using Acquire and Analysis 2.3 software (Physiologic Instruments, San Diego, CA). *I*_sc_ in response to forskolin alone (no modulator treatment) was considered as baseline activity (Δ*I*_sc−Fsk_). Cumulative changes of *I*_sc_ in response to forskolin and CFTR modulator were used as the measure of total CFTR-activated currents.

### Western blotting

Differentiated hNECs were lysed with TNI lysis buffer (0.5% Igepal CA-630, 50 mM Tris pH 7.5, 250 mM NaCl, 1 mM EDTA) (48) containing protease inhibitor cocktail (Roche 04693159001) for 30 min on ice. As previously described (30), lysates were sonicated using the Bioruptor Pico (Diagenode, Liège, Belgium) at 4°C for 20 cycles (30 s on, 30 s off). Lysates were then centrifuged at 14,000 rpm at 4°C for 20 min. A BCA Protein Assay Kit (Thermo Fisher Scientific 23225) was used to determine lysate protein concentration. Lysates were separated using a NuPAGE 3%–8% Tris-Acetate gel (Thermo Fisher Scientific EA0375BOX) at 100 V for 30 min, and then at 150 V until the separation finished. 67 μg of lysate was loaded per sample, except for the WT/WT sample which was loaded at 10 μg since signal saturation is experienced with higher quantities of WT/WT protein. Wet transfer (20 V for 1 h at RT) was used to transfer proteins onto a nitrocellulose membrane. To detect CFTR protein bands, the membrane was incubated with anti-CFTR antibody 596 (1 : 500; University of North Carolina, Chapel Hill and Cystic Fibrosis Foundation) at 4°C overnight. ECL Select detection reagent (Cytiva RPN2235) was used to visualise protein bands on the ImageQuant LAS 4000 (GE Healthcare, Chicago, IL). Calnexin, detected *via* anti-calnexin antibody (1 : 1,000; Cell Signalling Technology 2679), was used as the loading control. Protein band densitometry was performed using ImageJ (National Institutes of Health, Bethesda, MD). CFTR maturation in S945L/G542X hNECs treated with LUM/IVA, TEZ/IVA and ELX/TEZ/IVA was quantified by measuring the level of mature CFTR (band C) as a fold increase compared to mature CFTR from untreated S945L/G542X hNECs. All data were normalized to the calnexin loading control.

### Molecular dynamics

The CFTR protein model used for molecular dynamics simulations was based on the ATP-bound human CFTR cryogenic electron microscopy (cryo-EM) structure (PDB ID: 6MSM) (3). The construction of the CFTR protein system, included modelling part of the R domain, was described previously (31). In brief, the CFTR was embedded in a simplified cell membrane model containing pure 1-palmitoyl-2-oleoyl-sn-glycero-3-phosphocholine (POPC) lipids then solvated with 150 mM NaCl on both the cytoplasmic and periplasmic sides.

In this study, the software Gromacs v2021.1 (49) was used for all MD simulations. Molecular mechanics parameters were supplied *via* the CHARMM36m (50) force field with the following modifications for faster simulation performance: virtual site topologies (51) for all molecules while using MkVsite (52) to generate topologies for the ATP molecule, as well as parameter adjustments specific to lipids (53). The initial positions of all molecules were equilibrated for production runs in two phases: first *via* energy minimisation using a steepest descent algorithm, then followed by a 6 ns simulation where position-restraints on all non-hydrogen atoms were imposed and then gradually relaxed.

A series of 2 μs production MD simulations were carried out with three replicates each of WT and S945L-CFTR. To decrease computational expense, elevated temperatures of 350 K (77°C) were used to accelerate conformational transitions, in addition to simulations at physiological temperatures of 310 K (37°C). The use of elevated temperatures to study defects in mutant CFTR was validated previously (31). All measurements of Root Mean Square Deviation (RMSD) were calculated using the positions of alpha carbons in the 6MSM PDB structure (3) as a reference. Scripts may be found at https://github.com/miro-astore/mdanalysis_scripts and https://github.com/miro-astore/gromacs_scripts.

### Umbrella sampling protocols

Umbrella sampling is a computational technique which can be used to investigate the energy required to induce a conformational change in proteins. This is achieved using a strong virtual spring which pulls on the protein in a controlled manner. By measuring the force exerted by this virtual spring, the energy required to unfold parts of the protein can be calculated. This technique was used in this study to compare the energy required for misfolding of WT and S945L CFTR.

The energy values in Figure 3C were calculated by measuring the force profile as the amino acids Y852 and S/L945 were pulled away from each other. A virtual spring with a strength of 10 kcal/mol/Å^2^ was used to vary the distance between the alpha carbons of these amino acids from 6 Å to 16 Å. Umbrella sampling simulations were performed in 10 windows spaced 1 Å apart.

After 100 ns of equilibration, 100 ns of sampling was then collected for each window. The resultant energy profiles were calculated from the average of the five profiles determined from five independent samples of 20 ns each. Uncertainties for these results were calculated as twice the standard error of the mean for these five samples. Convergence of the umbrella sampling was determined through the observation that the uncertainties calculated from this method were below 1 kcal/mol across the energy surfaces of both WT- and S945l-CFTR.

Plumed 2.6 (54–56) was used to perform the umbrella sampling, and the WHAM code (57) was used to calculate the energy profiles. Umbrella sampling scripts may be found at https://github.com/miro-astore/plumed_scripts/.

### Statistical analysis

**Figure 1:**
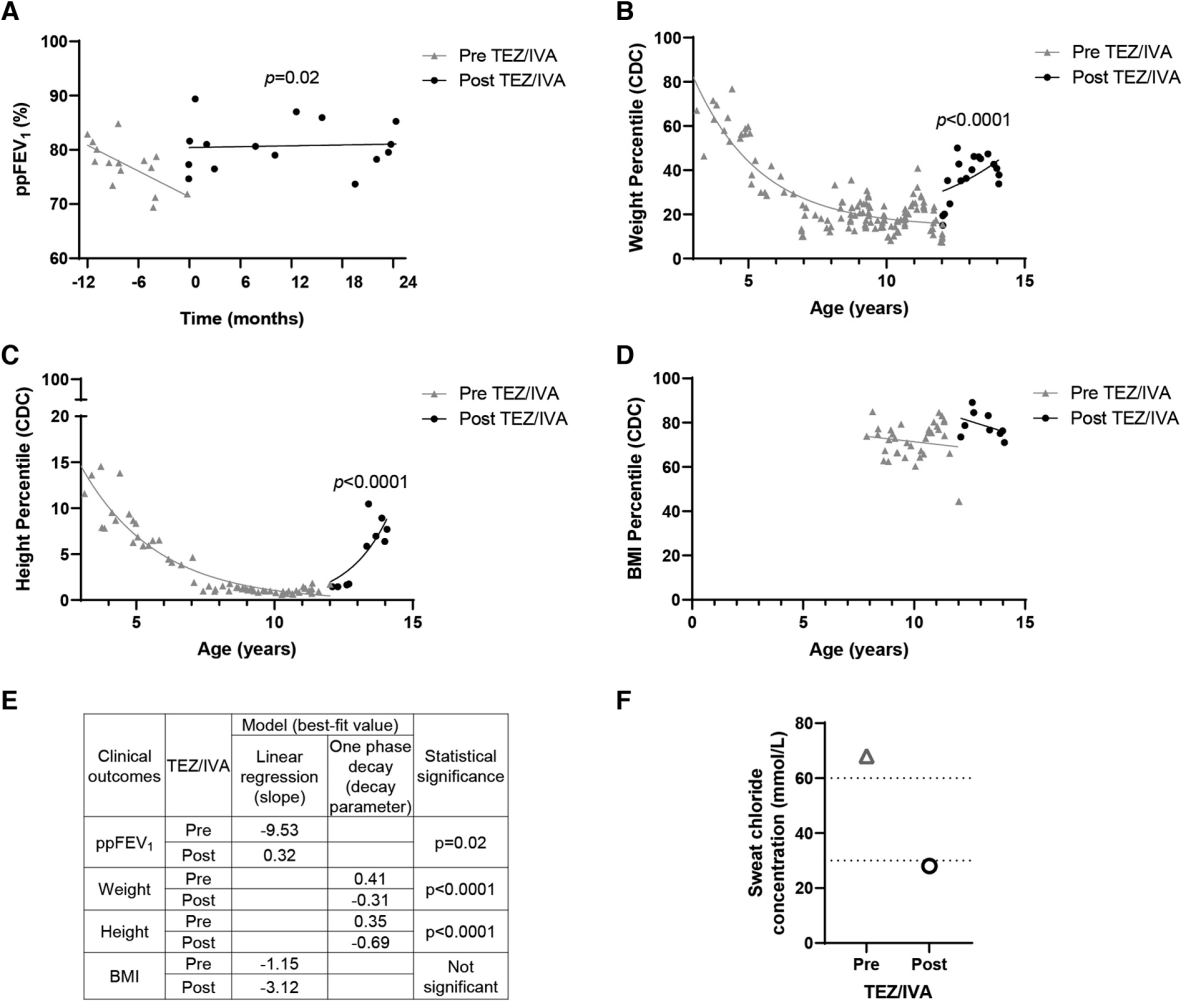
Effect of TEZ/IVA *in vivo* on lung function, nutritional factors and sweat chloride concentration in a patient with S945L/G542X-CFTR. (**A**) Lung function (ppFEV_1_) 12 months pre and 24 months post initiation of TEZ/IVA treatment. Serial (**B**) weight, (**C**) height and (**D**) body mass index (BMI) measurement percentiles from three years of age until 24 months post initiation of TEZ/IVA. Percentiles are based on data from the Centers for Disease Control and Prevention (CDC) growth charts. (**E**) Table summarizing statistical models applied to clinical outcome measurements and the best-fit values pre and post initiation of TEZ/IVA treatment. A simple linear regression was fit to the ppFEV_1_ (**A**) and BMI percentile (**D**) measurements from the time periods pre TEZ/IVA (grey line) and post TEZ/IVA (black line). The slope for each time period was estimated and the difference between them was tested. A one phase decay model was fit to the weight (**B**) and height (**C**) percentile measurements from the time periods pre TEZ/IVA (grey line) and post TEZ/IVA (black line), with zero as a shared plateau value and separate intercept values. The decay parameter for each time period was estimated and the difference between them was tested. (**F**) Sweat chloride concentration (mmol/L) measurements taken at baseline and 24 months post initiation of TEZ/IVA treatment. Dotted lines indicate lower limits of CF diagnostic (>60 mmol/L) and CF indeterminate (30–59 mmol/L) value ranges. No statistical modelling for sweat chloride concentration was performed due to single measurements pre and post TEZ/IVA.

**Figure 2:**
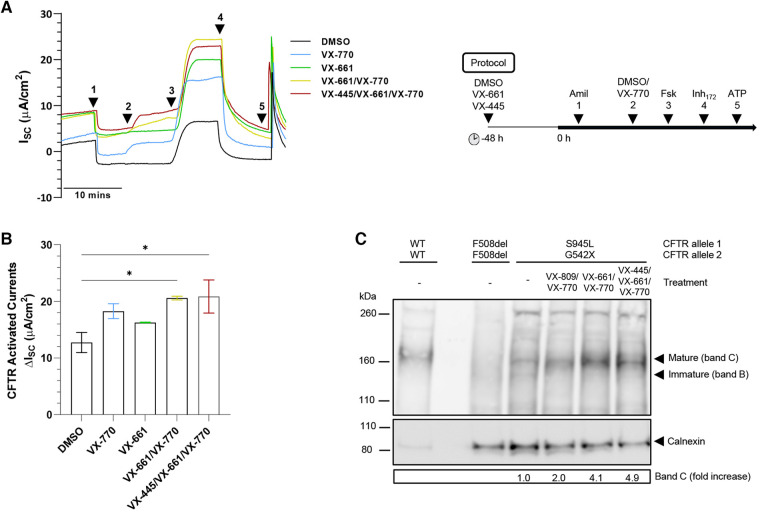
Effect of TEZ/IVA *in vitro* in S945L/G542X-CFTR patient-derived human nasal epithelial cell (hNEC) air-liquid interface (ALI) cultures. (**A**) Representative Ussing chamber recordings of short circuit current (*I*_SC_) in hNECs from a S945L/G542X participant. The protocol used to measure functional CFTR expression in hNECs pre-treated at 48 h with corrector (5 μM VX-661 or 3 μM VX-445/18 μM VX-661) or vehicle (0.01% DMSO), followed by sequential addition from 0 h with 100 μM apical amiloride (1. Amil), apical 0.01% DMSO or 10 μM VX-770 (2. DMSO, VX-770), 10 μM basal forskolin (3. Fsk), 30 μM apical CFTR-specific inhibitor (4. CFTR_Inh−172_) and 100 μM apical ATP (5. ATP). A basolateral-to-apical chloride gradient was used. (**B**) Bar graphs of total mean CFTR activated currents stimulated by DMSO or VX-770 plus Fsk in hNECs untreated or pre-treated with VX-661 or VX-445/VX-661. Data are from 2 to 3 independent ALI cultures and are represented as Mean ± SEM. Ordinary one-way ANOVA was used to determine statistical significance. *p < 0.05. (**C**) Western blot in cell lysates from S945L/G542X differentiated nasal epithelial cells. Cell lysates of F508del/F508del and WT/WT hNECs were used as controls to indicate protein size of CFTR bands B and C. To avoid signal saturation, the WT/WT sample was loaded at 7% of the mutant CFTR samples. S945L/G542X CFTR maturation following treatment with LUM/IVA (VX-809/VX-770), TEZ/IVA (VX-661/VX-770) or ELX/TEZ/IVA (VX-445/VX-661/VX-770) was quantified by measuring the level of mature CFTR (band C) in treated cells as a fold increase compared to mature CFTR in untreated cells. Band C represents the mature, complex-glycosylated CFTR. Band B represents the immature, core-glycosylated CFTR. All data were normalized to the calnexin loading control.

Statistical analysis advice was received from Stats Central, UNSW. Data for Figure 1 was fitted to a simple linear regression or one phase decay model. A simple linear regression was fit to the ppFEV_1_ (Figure 1A) and BMI (Figure 1D) percentile measurements from the time periods pre and post TEZ/IVA. The slope for each time period was estimated and the difference between them was tested. A one phase decay model was fit to the weight (Figure 1B) and height (Figure 1C) percentile measurements from the time periods pre and post TEZ/IVA, with zero as a shared plateau value and separate intercept values. The decay parameter for each time period was estimated and the difference between them was tested. Data for Figure 2 are presented as bar graphs with mean ± standard error of the mean (SEM). Ordinary one-way ANOVA with multiple comparisons was used to determine statistical significance. All statistical analysis was performed using GraphPad Prism v9.0.1 (GraphPad Software, San Diego, CA). A *p*-value of <0.05 was considered statistically significant.

## Results

### Improvement in *in vivo* lung function, sweat chloride and nutritional parameters

Having demonstrated restoration of S945L-CFTR activity *in vitro*, the *in vivo* effect of TEZ/IVA was assessed by reviewing the participant's clinical parameters. The mean ppFEV_1_ in the 12 months pre TEZ/IVA was 77.19 and improved to 80.79 in the 12 months post TEZ/IVA (Table 1). The absolute and relative changes in ppFEV_1_ at 12 months post TEZ/IVA compared to baseline (immediately pre TEZ/IVA) were 15.17 percentage points and 21.11%, respectively (Table 1). The slope of decline in lung function (ppFEV_1_) significantly (*p *= 0.02) changed in the 24 months post TEZ/IVA initiation, becoming positive (Figure 1A). Furthermore, there was an improvement in the trajectory of nutritional parameters, including weight (*p *< 0.0001, Figure 1B) and height percentiles (*p *< 0.0001, Figure 1C). The slope of change in BMI percentile was not significantly different after commencing treatment with TEZ/IVA (Figures 1D,E). In the 24 months pre TEZ/IVA initiation, the participant required seven admissions for optimisation of lung function, including treatment with intravenous antibiotics and reported one episode of pancreatitis (Table 1). In the 24 months post TEZ/IVA initiation, no admissions were required, and no episodes of pancreatitis were reported. Following TEZ/IVA initiation, the participant also experienced a 40 mmol/L decrease in sweat chloride concentration (indicating improvement in CFTR function). Sweat chloride concentration decreased from 68 to 28 mmol/L, dropping below both the CF diagnostic (>60 mmol/L) and CF indeterminate (30–59 mmol/L) value ranges (58) (Figure 1F).

**Table 1:** Summary of patient clinical outcomes 12 months pre and post TEZ/IVA.

	ppFEV1 (%)	Height percentile	Weight percentile	BMI percentile	Admissions	Sweat chloride (mmol/L)
12 months pre TEZ/IVA (mean)	77.19	1.32	18.34	71.38	4	–
Measurement at baseline	71.83	1.71	15.42	44.49	–	68
12 months post TEZ/IVA (mean)	80.79	1.58	33.79	81.48	0	–
Measurement at 12 months post TEZ/IVA	87.00	1.76	40.23	84.58	–	28*
Absolute change	15.17	0.05	24.81	40.09	–	−40
Relative change	21.11%	2.92%	160.89%	90.11%	–	−58.82%

ppFEV1, percent predicted forced expiratory volume in 1 s; BMI, body mass index.

Absolute and relative changes are calculated from baseline (last measurement pre TEZ/IVA). Percentiles are based on data from the Centers for Disease Control and Prevention (CDC) growth charts.

* Measurement performed 24 months post TEZ/IVA initiation.

### S945L-CFTR activity is significantly increased by TEZ/IVA in patient-derived nasal epithelial cells

Mature, differentiated S945L/G542X hNECs had intact junction integrity with transepithelial electrical resistance greater than 400 Ω.cm^2^ (Supplementary Figure S1A). To assess ion transport, short-circuit current (*I*_sc_) measurements were performed (Figure 2A). Epithelial sodium channel and calcium-activated chloride channel activity was unchanged by TEZ (Supplementary Figures S1B,C). S945L/G542X hNECs demonstrated baseline forskolin-activated *I*_sc_ (*I*_sc-Fsk_) of 12.73 ± 1.78 µA/cm^2^ (Figure 2B, Supplementary Table S1). Potentiation with IVA led to a 1.43-fold increase in *I*_sc-Fsk_, reaching 5.52 µA/cm^2^ above baseline, though statistical significance was not observed (*p *= 0.09; total *I*_sc-Fsk_: 18.25 ± 1.31 µA/cm^2^). TEZ monotherapy produced a 1.28-fold increase in *I*_sc-Fsk_, reaching 3.53 µA/cm^2^ above baseline, though statistical significance was not observed (*p *= 0.39, total *I*_sc-Fsk_: 16.26 ± 0.05 µA/cm^2^). Combination therapy with TEZ/IVA led to a significant (*p *= 0.02) 1.62-fold increase in *I*_sc-Fsk_, reaching 7.85 µA/cm^2^ above baseline (total *I*_sc-Fsk_: 20.58 ± 0.33 µA/cm^2^). CFTR-specific inhibitor (CFTR_Inh-172_) currents mirrored the trend observed in total CFTR-activated *I*_sc-Fsk_ (Supplementary Figure S1D, Supplementary Table S1). The triple therapy ELX/TEZ/IVA (total *I*_sc-Fsk_: 20.85 ± 2.93 µA/cm^2^) did not increase *I*_sc-Fsk_ beyond that which was recorded for dual therapy (Figure 2B, Supplementary Table S1).

### S945L-CFTR maturation is improved by TEZ/IVA in patient-derived nasal epithelial cells

CFTR protein expression and maturation was assessed in S945L/G542X hNECs with and without modulator treatment using Western blot (Figure 2C). Reference F508del/F508del and WT/WT hNECs were used to identify the location of immature, core-glycosylated CFTR at ∼130 kD (band B) and mature, complex-glycosylated CFTR at ∼160 kD (band C). In untreated S945L/G542X hNECs, the presence of immature and mature CFTR was detected at 10% and 90% of total CFTR protein, respectively. In S945L/G542X hNECs treated with TEZ/IVA, only mature CFTR (band C) was present, and had 4.1-fold higher abundance relative to the untreated S945L/G542X hNECs. Lysate from hNECs which were treated with either LUM/IVA or ELX/TEZ/IVA was also tested since LUM and ELX are known correctors of immature CFTR protein (59, 60). LUM/IVA and ELX/TEZ/IVA increased the levels of mature CFTR relative to the untreated S945L/G542X cells by 2.0- and 4.9-fold, respectively. The increase in mature CFTR with modulator treatment is consistent with the S945L/G542X hNEC functional rescue indicated by increased short-circuit current with TEZ/IVA (Figure 2B).

### *In silico* characterization of S945l-CFTR identifies misfolding of transmembrane helix 8 and disruption of the R domain

**Figure 3:**
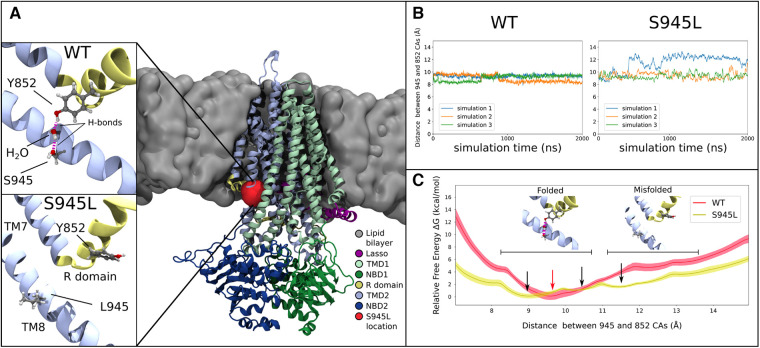
Molecular dynamics and free energy calculations to characterise the S945L mutation. (**A**) The open structure of the CFTR protein is used in all computational work (PDB ID: 6MSM), which is depicted here as coloured ribbons embedded within a grey lipid bilayer. A red blob shows the location of the S945 amino acid between TM8 and the R domain; this region is critical for channel gating functions. In simulations 1, 2 and 3 of WT-CFTR, a network of hydrogen bonds is found that connects Y852 to S945. This network is absent in simulations of S945L-CFTR, as the mutation replaces the polar serine side chain with a hydrophobic leucine side chain that is incapable forming hydrogen bonds. (**B**) The distance between S/L945 and Y852 alpha carbon atoms measured in three unbiased simulations of WT- and S945L-CFTR at 77°C (350 K). The distance trace is stable in WT-CFTR due to the formation of hydrogen bonds between Y852 and S945, where the alpha carbons of these amino acids remain at a distance of 9.1 ± 0.3 Å. Distances are denoted here by Mean ± SD. Some destabilisation is seen in S945L-CFTR with unbiased MD: in simulations 2 (orange) and 3 (green) the average distance is 9.4 ± 0.5 Å, while in simulation 1 (blue line), a conformational transition occurs after approximately 400 ns, that increases the average distance to 12.0 ± 0.6 Å. (**C**) Relative free energy as the distance between S/L945 and Y852 is varied in umbrella sampling calculations. In WT-CFTR there exists one deep free energy minimum at 9.5 Å (red arrow). In S945L-CFTR there are instead three shallow free energy minima between 9.0 Å and 13.0 Å (black arrows), separated by small barriers with heights of 2.0 kcal/mol or less. Black horizontal bars indicate regions of two distinct local conformations: one at distances below 10.8 Å, where the R domain and TM8 are properly folded and a Y852-S945 hydrogen bond network exists; the other at distances above 11.2 Å, where the R domain and TM8 separate into a misfolded conformation. The relative free energies obtained through umbrella sampling suggest that such misfolding is more likely to occur in S945L-CFTR.

We next characterized the structural defect of S954L-CFTR using MD simulations. The WT 6MSM model (3) was mutated to S945L. The S945 amino acid is located in TM8, near the membrane surface on the cytoplasmic side of CFTR (Figure 3A). Three 2 μs simulations were performed. The simulations demonstrated that S945 plays a structural role by bridging TM8 with adjacent TM7 and R domain helices. This is facilitated by hydrogen bonding to Y852 in the R domain. Two configurations of this hydrogen bonding were observed in WT-CFTR simulations, indirect hydrogen bonding *via* a mediating water molecule (State 1), or direct hydrogen bonding between S945 and Y852 (State 2) (Supplementary Figure S2A). Both states keep Y852 and S945 in proximity to one another (Supplementary Figure S2B). The interactions were stable throughout three replicate 2 μs MD simulations of WT-CFTR, at both physiological temperature (37°C; Supplementary Figure S3) and an elevated temperature (77°C; Figure 3B, Supplementary Figure S4).

In S945L-CFTR, the mutant leucine amino acid (L) lacked the ability of WT serine (S) to form sidechain hydrogen bonds (Figure 3A). The effects of this change were measured and compared in WT and S945L-CFTR. Compared to WT-CFTR, S945L-CFTR had increased conformational fluctuation in the R domain elbow, as measured by root mean square deviation (RMSD, Supplementary Figure S4). This suggested that the S945L mutation decreased R domain stability. The instability and loss of the hydrogen bond anchor enabled the R domain Y852 sidechain in one S945L simulation to physically rotate away from TM8 (Figure 3B). Since the R domain is critical for protein gating, these observed movements and destabilization of the R domain suggest that S945L-CFTR is likely to possess defective gating function.

In S945L-CFTR, movement of the R domain away from its folded conformation was observed in only one of three replicate simulations at 77°C (Figure 3B). This movement was not observed in any of three replicates at physiological temperature (37°C, Supplementary Figure S3B). These results suggested that unbiased MD was not sufficient to reliably explore potential pathological changes in S945L-CFTR due to the slow movements of the R domain. These slow movements motivated further investigation into the energetics of CFTR motion by a rigorous method known as umbrella sampling. This involved using a virtual spring to pull apart the alpha carbon atoms of amino acids S/L945 and Y852. As the atoms were pulled away from each other, the force exerted on the spring was measured for calculation of the energy required to break the link between amino acid S/L945 and Y852.

Umbrella sampling calculations of the WT-CFTR free energy surface confirmed the stability of the S945-Y852 hydrogen bond (Figure 3C). The WT-CFTR free energy surface featured a deep local minimum when Y852 and S945 alpha carbon atoms were separated by 9.5 Å. Distances greater than 12 Å were inhibited in WT-CFTR by high energies of 4.7 kcal/mol, which would not be easily accessible at physiological temperatures. Therefore, the most likely state for WT-CFTR to occupy was the deep minimum at 9.5 Å, consistent with the observed distance between 945 and 852 alpha carbon atoms in three replicate 2 μs MD simulations of WT-CFTR (Figure 3B). This indicates the constraining effect of the hydrogen bond between S945 and Y852. The steepness of the local minimum was also consistent with the smaller fluctuations observed during the unbiased MD simulations, as if bound by a tight spring (Figure 3B). In contrast, the S945L-CFTR free energy surface showed several shallow local minima between the near-native 9.0 Å and maximum distance of 13 Å (Figures 3B,C). This free energy surface correlates with the larger fluctuations observed in the unbiased simulations, as if bound by a weaker spring than WT-CFTR. The small, intervening free energy barriers of 2.0 kcal/mol or less would likely be overcome at physiological temperatures. This same amount of energy in WT-CFTR would only allow the exploration of a limited region, between 8.9 and 10.6 Å. This indicated that S945L-CFTR was more likely to spontaneously transition into misfolded conformational states where the R domain elbow, containing Y852, moves away from TM8. Further, the altered energy landscape in Figure 3C means that it is likely that the overall protein folding energy of S945L-CFTR will be different to WT-CFTR, which may give rise to a folding defect in the mutant (61).

## Discussion

We have described patient-specific baseline CFTR function and response to CFTR-modulating drugs in nasal epithelial cell models derived from a CF individual with S945L/G542X-CFTR. Following initiation of TEZ/IVA therapy by the participant, we compared their lung function, sweat chloride concentration and nutritional parameters pre and post treatment. Furthermore, we complemented our functional studies of S945L-CFTR with molecular dynamics (MD) simulations to understand the functional and structural changes in CFTR caused by the S945L mutation.

S945L-CFTR has been described previously *via* biochemical studies. Low (<10%) band C expression of CFTR relative to WT has been reported *via* western blot using Chinese hamster ovary (CHO) cells (38). Furthermore, band C CFTR expression of 42% of WT level was described in Fischer rat thyroid cells (36). In this study, 90% of total CFTR protein (band B + C) in untreated S945L/G542X hNECs was detected to be mature CFTR (band C), indicating presence of fully processed CFTR that has passed through the Golgi apparatus. S945L-CFTR localisation to the cell surface has been evidenced previously wherein single-channel patch clamp studies were performed in S945L-CFTR-expressing CHO cells, and demonstrated reduced channel mean open probability measurements compared to WT-CFTR (38). S945L-CFTR baseline function *via* CFTR-mediated chloride transport in patient-derived differentiated hNECs (*I*_sc-Fsk_: 12.73 ± 1.78 µA/cm^2^) was approximately similar to R352Q-CFTR activity (*I*_sc-Fsk_: 14.8 ± 1.4 µA/cm^2^), which we previously characterised as having a conductance defect (30). Together, this suggests that S945L-CFTR is localised to the cell surface and has residual channel function.

Functional assessment of S945L-CFTR activity in Ussing chambers demonstrated a 1.43-fold increase in activity upon IVA treatment compared to baseline. This suggests that S945L causes a CFTR gating defect that can be restored with IVA monotherapy. However, the treatment benefit derived from IVA is limited by the number of S945L-CFTR channels available for potentiation at the plasma membrane, and it is likely that a ceiling effect is reached. In a previous study of short-circuit current in S945L-expressing FRT cells, S945L was shown to have a much greater 12.5-fold increase in activity with IVA, increasing from 6% to 75% of WT activity (36). An *in vivo* study of CFTR-dependent sweat secretion in two CF patients with S945L/G542X and S945L/F508del genotypes also demonstrated the responsiveness of S945L to IVA reporting that IVA restored near-normal function to S945L-CFTR and supporting the use of IVA as a treatment for CF patients with this allele (62). If the number of S945L channels at the plasma membrane were increased by a corrector compound, then more defective channels available for potentiator-mediated restoration may result in increased CFTR activity.

S945L is reported to have a protein maturation defect (38). Although functional characterisation of S945L-CFTR has been performed *via* molecular studies in heterologous expression systems and described to interfere with protein folding (36, 38), this has not been validated in primary cells. Furthermore, the results from these studies were not in agreement as to the severity of the folding defect, and no functional testing was conducted to validate these findings. Functional assessment of S945L-CFTR *via* short-circuit current measurements in this study showed a 1.28-fold increase in CFTR activity in response to TEZ monotherapy. Although statistical significance was not achieved, this increase in CFTR activity may indicate that TEZ rescues misfolded S945L-CFTR. This is supported by the observation *via* western blot that 10% of total CFTR in untreated hNECs was immature CFTR (band B) which suggests that S945L-CFTR complex glycosylation was impeded. Upon treatment with LUM/IVA or TEZ/IVA, band B immature CFTR was no longer present, and an increase in mature CFTR (band C) of at least 2.0-fold was observed. This supports the effectiveness of CFTR corrector(s) in correcting misfolded protein and chaperoning it to the cell surface. We reasoned it is likely that S945L-CFTR function was not significantly increased by TEZ monotherapy when CFTR-mediated ion transport was measured in Ussing chambers because the CFTR rescued to the cell surface sustained a channel gating defect.

Like the prototypical folding mutation F508del, S945L has been reported to cause multiple defects including protein maturation, channel gating and conductance (38). It is unsurprising then that the results of the present study found a significant 1.62-fold increase in S945L-CFTR function following combination therapy with TEZ/IVA. This suggests that S945L produces both CFTR folding and gating defects that are amenable to CFTR modulator combination therapy. This is in support of previous molecular studies of S945L (38), yet it is the first study to test combination therapy for S945L *in vitro*. Furthermore, the participant showed evidence of significant clinical benefit with TEZ/IVA treatment. Lung function (ppFEV_1_), sweat chloride concentration, and height and weight percentiles were improved with TEZ/IVA. Although the rate of change in BMI percentile was unchanged with TEZ/IVA treatment, it is worth noting that neither was any significant effect on BMI observed in the Phase III trial evaluating TEZ/IVA in patients 12 years and older homozygous for F508del (63).

Since the approval of TEZ/IVA for S945L, the triple combination modulator therapy ELX/TEZ/IVA has expanded treatment access to more than 90% of people with CF and is currently the most effective treatment for improving patient lung function (19, 64). Additionally, the potential utility of novel co-potentiators (65, 66), correctors (67–69), amplifiers (70), and a drug targeting the misfolding detection machinery (71) have been assessed. Since the participant enrolled in this study was receiving treatment with TEZ/IVA, the focus was to compare the participant's *in vitro* response to TEZ/IVA with their *in vivo* response to TEZ/IVA*.* The correlation in the participant's drug response *in vitro* and *in vivo* supports the use of patient-derived cells for predicting patient-specific treatment response. As new therapeutic options become available and as patients receive access to multiple therapies, it will be important to determine which drug is most effective for the patient. Since the S945L mutation is approved for treatment with ELX/TEZ/IVA by the FDA (43), it was expected that ELX/TEZ/IVA would produce some improvement in CFTR function. The participant's *in vitro* response to ELX/TEZ/IVA was approximately equal to that of TEZ/IVA. This suggests that ELX/TEZ/IVA may achieve a similar improvement in *in vivo* clinical outcomes for this participant as that observed upon TEZ/IVA treatment. The importance of assessing drug response on an individualised basis is emphasised by the fact that variable clinical responsiveness has been observed in clinical trials with CFTR modulators, even amongst patients with the same *CFTR* mutation (17, 72). Our study adds evidence to the increasing number of *n* = 1 studies showing that *in vitro* functional assessment of CFTR activity in patient-derived cell models correlates with *in vivo* patient outcomes (22–28), supporting the use of such models to advance the approval of therapeutics in the future.

In addition to functionally characterizing S945L *via* response to CFTR modulators, we also used *in silico* protein modelling *via* MD simulations to determine the molecular cause of both a folding and a gating defect in S945L-CFTR. In MD simulations of WT-CFTR, a stable network of hydrogen bonds linking Y852 in the R domain to S945 on TM8 was identified. This interaction was not visible in the cryo-EM structure of the channel, where S945 faced away from Y852 (3), demonstrating the utility of MD simulations for elucidating the molecular details of protein function. In simulations of S945L-CFTR, this interaction was disrupted, and our results indicate that the R domain may move away from TM8. Due to the importance of the R domain in gating, we expect that a change to its position could lead to defective channel gating. Simultaneously, the disruption of this hydrogen bond link between two different domains is likely to give rise to a folding defect, as the elbow region will be less likely to fold correctly, and the overall folding energy of the protein is altered. Hence, our MD simulations have revealed a unique mode of pathogenesis for the S945L mutation that also provides a greater understanding of CFTR function. This is another example in a set of MD simulations of the CFTR protein which suggest that CFTR modulators can elicit a response in multiple *CFTR* missense mutations with unique molecular phenotypes (30–33).

This study provides evidence that the combination of TEZ/IVA significantly increases S945L-CFTR activity in this participant's differentiated nasal cells, beyond the response to IVA alone. While S945L was initially approved for IVA, it is clear that the approval for TEZ/IVA combination therapy was appropriate, as supported by data from this study. The *in vitro* drug response correlated with the participant's *in vivo* clinical benefit following initiation of TEZ/IVA therapy. This highlights the importance of *n* = 1 clinical data used together with patient-derived cell model data, to predict CFTR drug response and evaluate the effect of novel therapeutics on CFTR function on an individualised basis. Furthermore, our study showed *in vitro* and *in silico* that S945L causes both folding and gating defects in the CFTR protein. Characterisation of the functional and structural defects of S945L-CFTR increases our understanding of CFTR folding and gating regulation and provides a potential pathway to expand further drug access to CF patients in the future.

## Data Availability

The raw data supporting the conclusions of this article will be made available by the authors, without undue reservation.
