# Supplementary material for: S945L-CFTR molecular dynamics, functional characterization and tezacaftor/ivacaftor efficacy in vivo and in vitro in matched pediatric patient-derived cell models
Source: Front Pediatr. 2022 Nov 16;10:1062766. doi: 10.3389/fped.2022.1062766 (PMC9709344; doi:10.3389/fped.2022.1062766)
Supplement: Supplementary file 1 [file Datasheet1.docx]

Supplementary Material

# Supplementary Figures and Tables

## Supplementary Figures


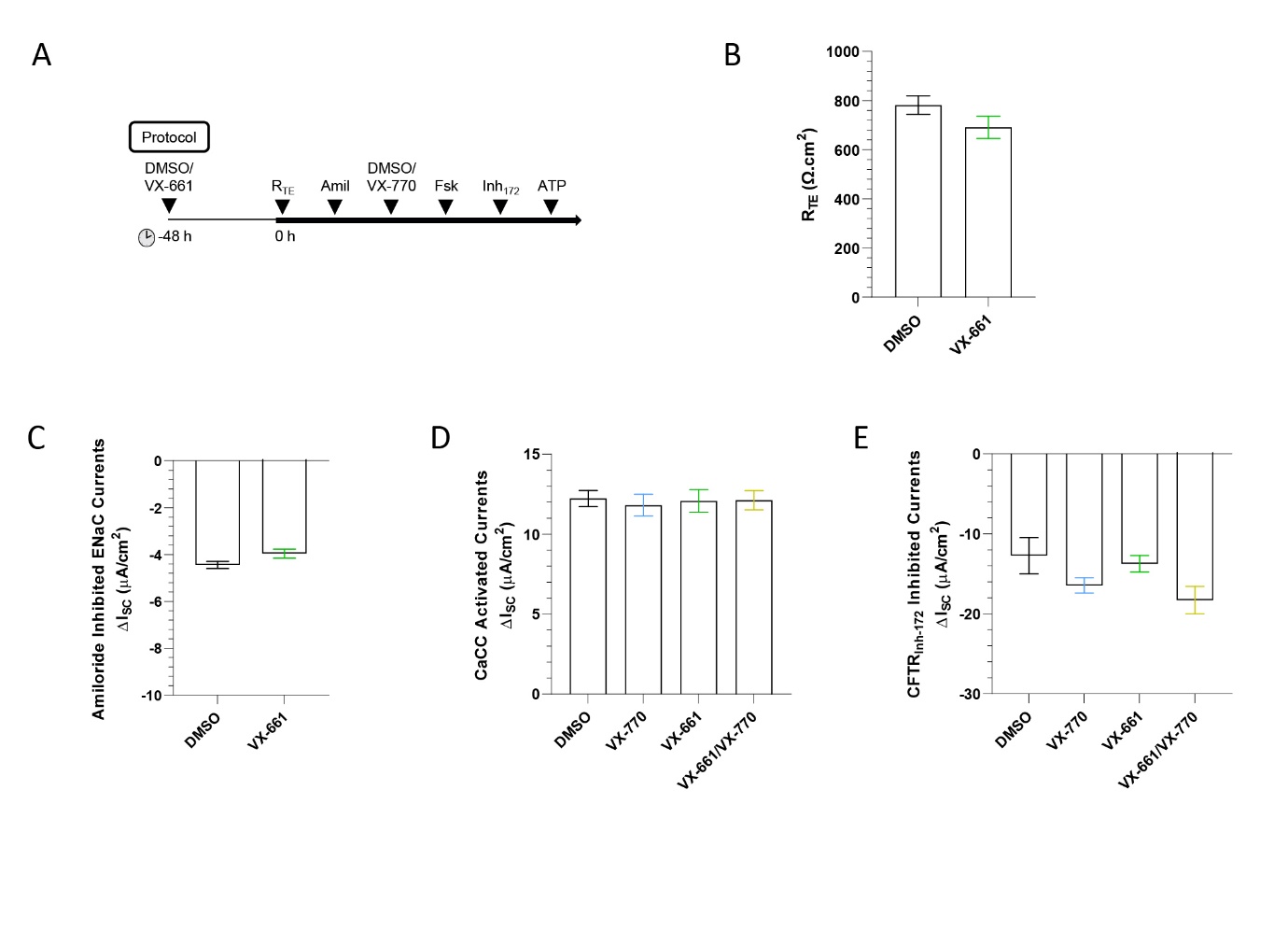


**Supplementary Figure 1. Effect of TEZ/IVA on epithelium integrity and short circuit currents in S945L/G542X-CFTR patient-derived human nasal epithelial cell (hNEC) air-liquid interface (ALI) cultures. (A)** Protocol indicating pre-treatment with VX-661 or DMSO (vehicle) at 48 h, then at 0 h, measurement of transepithelial electrical resistance (R_TE_), followed by sequential addition of amiloride, VX-770 or DMSO (vehicle), forskolin, CFTR_Inh-172_ and ATP. Bar graphs of mean **(B)** R_TE_ (Ω.cm^2^), **(C)** amiloride-inhibited epithelial sodium channel (ENaC) currents, **(D)** ATP-activated calcium-dependent chloride channel (CaCC) currents and **(E)** CFTR-inhibited (CFTR_Inh-172_) currents. Data represents mean current in ALI cultures pre-treated with VX-661 or DMSO, and stimulated by VX-770 or DMSO plus Fsk (D-E only). Data are represented as Mean ± SEM. Unpaired t test (B-C) or ordinary one-way ANOVA with multiple comparisons (D-E) were used to determine statistical significance. Refer to Supplementary Table 1 for short-circuit current values.


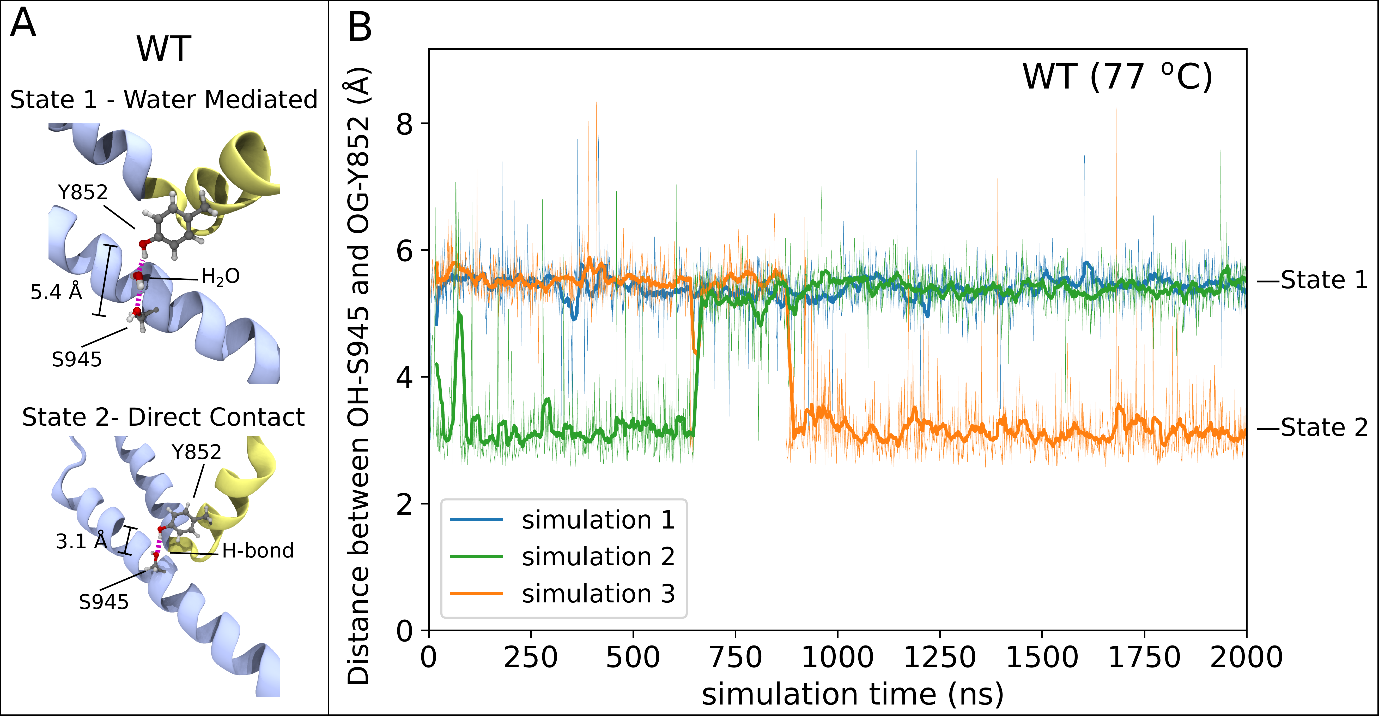


**Supplementary Figure 2.** **A closer inspection of the hydrogen bonds between Y852 and S945 in WT-CFTR. (A)** The simulations show two possible arrangements of the hydrogen bond network connecting Y852 and S945. State 1 involves a mediating water molecule that forms twos hydrogen bonds, respectively to each side chain of S945 and Y952. State 2 does not involve a mediating water molecule, so a single hydrogen bond is formed directly between S945 and Y952. **(B)** The distance between the oxygen atoms in the polar side chains of S945 and Y852 throughout the WT simulations at 77 °C. Three replicates of 2 µs each were conducted. In simulation 1 (blue), the system remains in state 1 throughout all 2 µs of the MD simulation. Simulation 2 begins in state 1, but transitions to state 2 after 900 ns. Simulation 3 begins in state 2, but transitions to state 1 after 700 ns. Data is graphed as a 20 ns moving average to clearly delineate the difference between the two states.


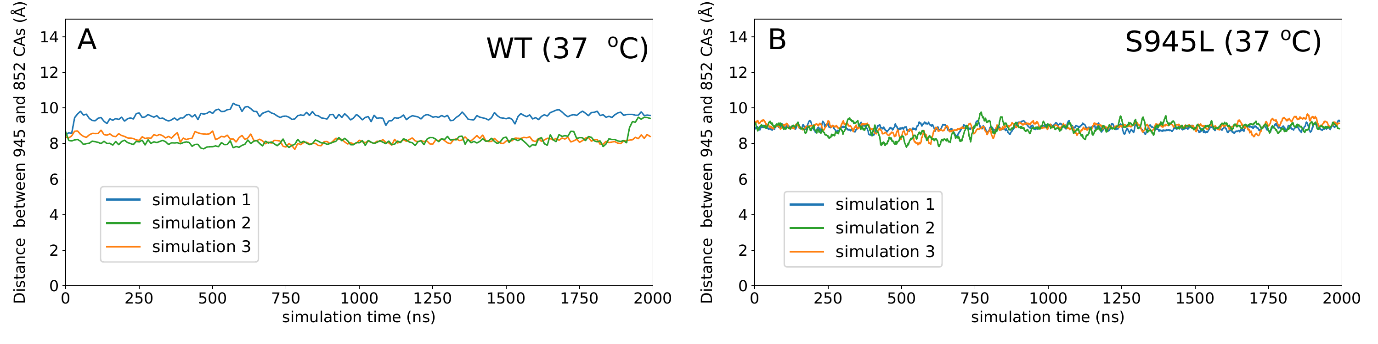


**Supplementary Figure 3. Distance between S945 and Y852 alpha carbon atoms from unbiased simulations of WT- and S945L-CFTR at 37 °C.** Three 2 μs simulations were conducted each for WT-CFTR and S945L-CFTR. **(A)** Two transitions from state 2 to state 1 is seen in WT-CFTR: within 100 ns for simulation 1, and after 1900 ns for simulation 2. Simulation 3 remains in state 2 throughout. **(B)** A single state is seen in S945L-CFTR with no significant perturbations at physiological temperatures. The alpha carbons of the Y852 and L945 amino acids remained at a distance between state 1 and state 2 of WT-CFTR. This suggests that misfolded conformations found in 77 °C simulations (Figure 3) are not feasibly reached within 2 μs of simulation time at 37 °C.


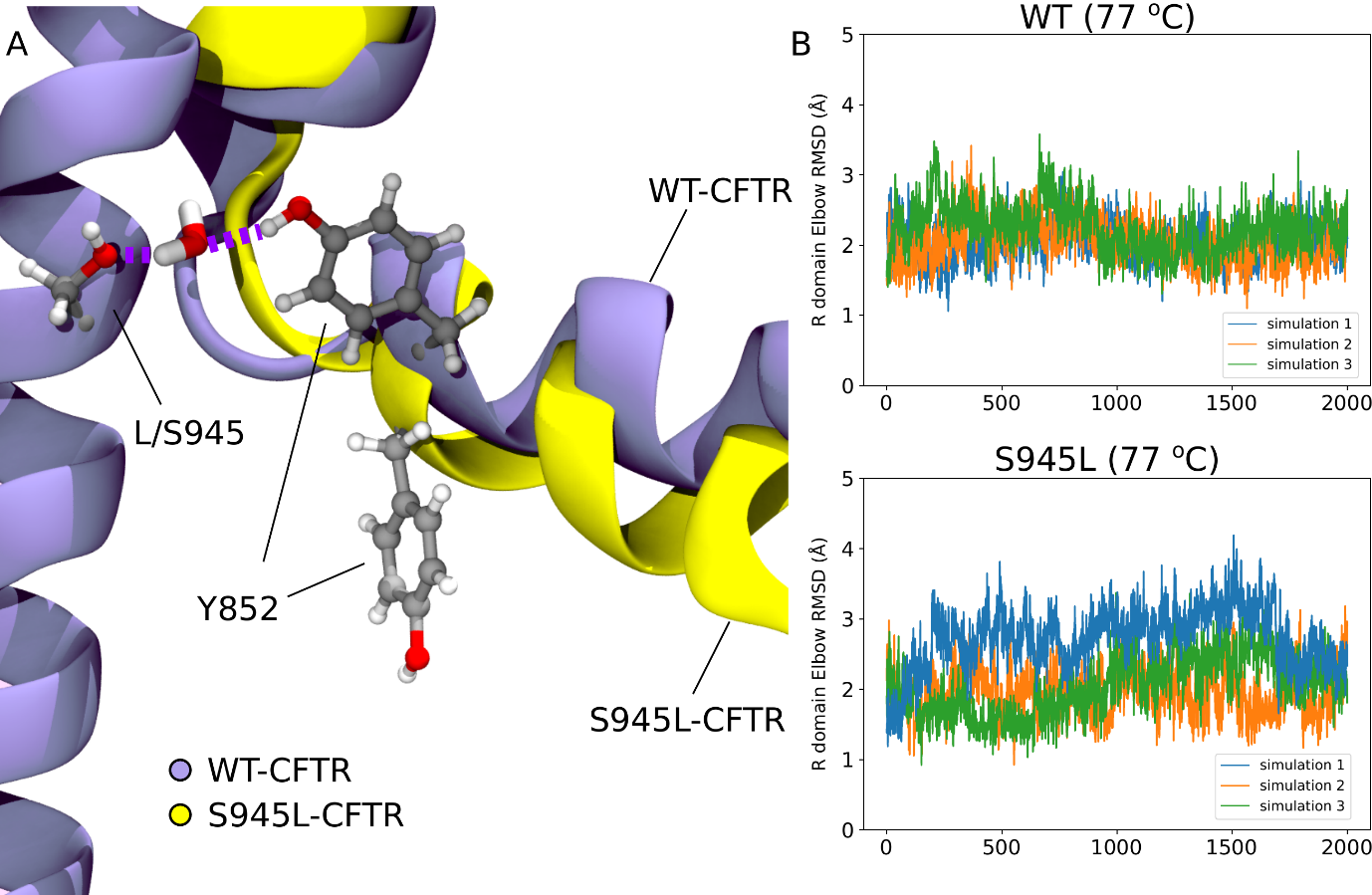


**Supplementary Figure 4.** **The local conformational change of the elbow between TM8 and the R domain.** **(A)** The S945L mutation in TM8 is located in proximity to Y852, which resides on the elbow of the R domain (amino acids 845-886). In WT-CFTR (yellow ribbons), a hydrogen bond network may form between S945 and Y852. This is not possible for S945L-CFTR (purple ribbons). **(B)** The Root Mean Square Deviation (RMSD) values of the elbow region of the R domain, calculated with reference to the alpha carbon atoms of the 6MSM CFTR structure at 77 ^o^C. Three 2-μs simulations were conducted for WT-CFTR as controls, where RMSD values fluctuate around 2 Å. Three additional 2 μs simulations were conducted for S945L-CFTR, in which simulation 1 displayed an increase of RMSD values to 3 Å, reflecting a destabilisation of the elbow region (blue). RMSD values of simulations 2 (orange) and 3 (green) do not display a destabilistaion. This is consistent with the observations of Figure 3B.

## Supplementary Tables

**Supplementary Table 1. Data for the short-circuit currents and electrophysiological parameters in patient-derived differentiated hNECs.**

|  | R_TE_ | ΔAmil | ΔATP | ΔCFTR-Activated | ΔCFTR_Inh-172_ |
| --- | --- | --- | --- | --- | --- |
| DMSO | 813.7 ± 70.23 | -4.67 ± 0.25 | 7.45 ± 0.20 | 12.73 ± 1.78 | -12.74 ± 2.25 |
| VX-770 | 748.7 ± 32.54 | -4.22 ± 0.09 | 6.61 ± 0.41 | 18.25 ± 1.31 | -16.45 ± 0.95 |
| VX-661 | 731.0 ± 13.75 | -4.08 ± 0.32 | 8.08 ± 0.13 | 16.26 ± 0.05 | -13.74 ± 1.05 |
| VX-661/  VX-770 | 651.3 ± 92.55 | -3.85 ± 0.29 | 6.32 ± 0.45 | 20.58 ± 0.33 | -18.29 ± 1.72 |
| VX-445/ VX-661/ VX-770 | 657.0 ± 103.00 | -3.31 ± 0.47 | 10.81 ± 0.31 | 20.85 ± 2.93 | -16.71 ± 0.71 |

Data presented are transepithelial resistance (R_TE_) and short-circuit current values for amiloride inhibited ENaC currents (ΔAmil), ATP-activated currents (ΔATP), CFTR-activated currents (ΔCFTR-Activated) and CFTR-Inh_172_ inhibited currents (ΔCFTR-Inh_172_). Data are represented as Mean ± SEM.
